# Tan’s two-body contact across the superfluid transition of a planar Bose gas

**DOI:** 10.1038/s41467-020-20647-6

**Published:** 2021-02-03

**Authors:** Y.-Q. Zou, B. Bakkali-Hassani, C. Maury, É. Le Cerf, S. Nascimbene, J. Dalibard, J. Beugnon

**Affiliations:** Laboratoire Kastler Brossel, Collège de France, CNRS, ENS-PSL Research University, Sorbonne Université, 11 Place Marcelin Berthelot, 75005 Paris, France

**Keywords:** Atomic and molecular collision processes, Ultracold gases

## Abstract

Tan’s contact is a quantity that unifies many different properties of a low-temperature gas with short-range interactions, from its momentum distribution to its spatial two-body correlation function. Here, we use a Ramsey interferometric method to realize experimentally the thermodynamic definition of the two-body contact, i.e., the change of the internal energy in a small modification of the scattering length. Our measurements are performed on a uniform two-dimensional Bose gas of ^87^Rb atoms across the Berezinskii–Kosterlitz–Thouless superfluid transition. They connect well to the theoretical predictions in the limiting cases of a strongly degenerate fluid and of a normal gas. They also provide the variation of this key quantity in the critical region, where further theoretical efforts are needed to account for our findings.

## Abstract (web summary)

Here the authors use Ramsey interferometry to study Tan’s contact in uniform two-dimensional Bose gas of 87Rb atoms across the Berezinskii–Kosterlitz–Thouless superfluid transition. They find that the two-body contact is continuous across the critical point.

## Introduction

The thermodynamic equilibrium of any homogeneous fluid is characterized by its equation of state. This equation gives the variations of a thermodynamic potential, e.g., the internal energy *E*, with respect to a set of thermodynamics variables such as the number of particles, temperature, size, and interaction potential. All items in this list are mere real numbers, except for the interaction potential whose characterization may require a large number of independent variables, making the determination of a generic equation of state challenging.

A considerable simplification occurs for ultra-cold atomic fluids when the average distance between particles *d* is much larger than the range of the potential between two atoms. Binary interactions can then be described by a single number, the s-wave scattering length *a*. Considering *a* as a thermodynamic variable, one can define its thermodynamic conjugate, the so-called Tan’s contact^1–9^

1 $$C\equiv \frac{8\pi m{a}^{2}}{{\hslash }^{2}}\ \frac{\partial E}{\partial a},$$

where the derivative is taken at constant atom number, volume, and entropy, and *m* is the mass of an atom. For a pseudo-spin 1/2 Fermi gas with zero-range interactions, one can show that the conjugate pair (*a*, *C*) is sufficient to account for all possible regimes for the gas, including the strongly interacting case *a* ≳ *d*^10,11^. For a Bose gas, the situation is more complicated: formally, one needs to introduce also a parameter related to three-body interactions, and in practice, this three-body contact can play a significant role in the strongly interacting regime^12–15^.

Since the pioneering experimental works of refs. ^16,17^, the two-body contact has been used to relate numerous measurable quantities regarding interacting Fermi gases: the tail of the momentum distribution, short-distance behavior of the two-body correlation function, radio-frequency spectrum in a magnetic resonance experiment, etc. (see refs. ^18,19^ and references therein). Its generalization to low-dimensional gases has also been widely discussed^13,20–28^. For the Bose gas case of interest here, experimental determinations of two- and three-body contacts are much more scarce, and concentrated so far on either the quasi-pure BEC regime^29,30^ or the thermal one^29,31^. Here, we use a two-pulse Ramsey interferometric scheme to map out the variations of the two-body contact from the strongly degenerate, superfluid case to the non-degenerate, normal one.

We operate with a uniform, weakly interacting two-dimensional (2D) Bose gas where the superfluid transition is of Berezinskii–Kosterlitz–Thouless (BKT) type^32,33^. For our relatively low spatial density, effects related to the three-body contact are negligible and we focus on the two-body contact. It is well known that for the BKT transition, all thermodynamic functions are continuous at the critical point, except for the superfluid density^34^. Our measurements confirm that the two-body contact is indeed continuous at this point. We also show that the (approximate) scale invariance in 2D allows us to express it as a function of a single parameter, the phase-space density ${\mathcal{D}}=n{\lambda }^{2}$, where *n* is the 2D density, $\lambda ={(2\pi {\hslash }^{2}/m{k}_{{\rm{B}}}T)}^{1/2}$ the thermal wavelength, and *T* the temperature. Our measurements around the critical point of the BKT transition provides an experimental milestone, which shows the limits of the existing theoretical predictions in the critical region.

## Results

Our ultra-cold Bose gas is well described by the Hamiltonian $\hat{H}$, sum of the kinetic energy operator, the confining potential, and the interaction potential ${\hat{H}}_{{\rm{int}}}=a\hat{K}$ with

2 $$\hat{K}=\frac{2\pi {\hslash }^{2}}{m}\int \int {\hat{\psi }}^{\dagger }({\bf{r}})\ {\hat{\psi }}^{\dagger }({\bf{r}}^{\prime} )\ \hat{\delta }({\bf{r}}-{\bf{r}}^{\prime} )\ \hat{\psi }({\bf{r}}^{\prime} )\ \hat{\psi }({\bf{r}})\,{d}^{3}r\ {d}^{3}r^{\prime} .$$

Here $\hat{\delta }({\bf{r}})$ is the regularized Dirac function entering in the definition of the pseudo-potential^35^ and the field operator $\hat{\psi }({\bf{r}})$ annihilates a particle in **r**. Using Hellmann–Feynman theorem, one can rewrite the contact defined in Eq. (1) as $C=8\pi m{a}^{2}\langle \hat{K}\rangle /{\hslash }^{2}$.

In our experiment, the gas is uniform in the horizontal *xy* plane, and it is confined with a harmonic potential of frequency *ω*_*z*_ along the vertical direction. We choose ℏ*ω*_*z*_ larger than both the interaction energy and the temperature, so that the gas is thermodynamically two-dimensional (2D). On the other hand, the extension of the gas ${a}_{z}={(\hslash /m{\omega }_{z})}^{1/2}$ along the direction *z* is still large compared to the 3D scattering length *a*, so that the collisions keep their 3D character^36^. Therefore, the definition (1) of the contact and the expression (2) of the interaction potential remain relevant, and the interaction strength is characterized by the dimensionless parameter $\tilde{g}=\sqrt{8\pi }a/{a}_{z}\approx 0.16$.

If the zero-range potential $\hat{\delta }({\bf{r}}-{\bf{r}}^{\prime} )$ appearing in (2) did not need any regularization, the contact $C$ would be equal simply to $g_{2}(0) C_{0}$ where 

3 $${C}_{0}\equiv 4{(2\pi )}^{3{/}2}\frac{{a}^{2}{\bar{n}}N}{{a}_{z}}$$

sets the scale of Tan’s contact, with ${\bar{n}}$ the average 2D density and *N* the atom number. The in-plane two-body correlation function is defined by ${g}_{2}({\bf{r}})=\langle :\hat{n}({\bf{r}})\hat{n}(0):\rangle {/}\bar{{n}}^{2}$, where $\hat{n}({\bf{r}})$ is the operator associated with the 2D density and the average value is calculated after setting the particle creation and annihilation operators in normal order. We recall that for an ideal Bose gas, the value of *g*_2_(0) varies from 2 to 1 when one goes from the non-condensed regime to the fully condensed one^37^.

It is well known that *g*_2_(0) is generally an ill-defined quantity for an interacting fluid. For example, in a Bose gas with zero-range interactions, one expects *g*_2_(*r*) to diverge as 1/*r*^2^ in 3D and ${(\mathrm{log}\,r)}^{2}$ in 2D when *r* → 0^12,13^. On the other hand, when one properly regularizes the zero-range potential $\hat{\delta }$ in Eq. (2), Tan’s contact is well-behaved. In the zero-temperature limit, the mean-field energy of the 2D gas is $E=({\hslash }^{2}/2m)\tilde{g}\bar{n}N$^38^, leading to *C* = *C*_0_. In the large temperature, non-degenerate limit (but still assuming the s-wave scattering regime), one can use the virial expansion (see Supplementary Note 4 and ref. ^35^) to calculate the deviation of the free energy *F*(*N*, *A*, *T*, *a*) of a uniform quasi-2D gas with *N* atoms in an area *A* with respect to the ideal classical (Boltzmann) gas value. It reads $F-{F}_{{\rm{Boltzmann}}}=({\hslash }^{2}/m)\tilde{g}\bar{n}N$, from which the value of the contact *C* = 2*C*_0_ is obtained using *C* = (8*πma*^2^/ℏ^2^)(∂*F*/∂*a*)_*N*,*A*,*T*_.

In this work, we determine the contact experimentally by measuring the change in energy per atom *h*Δ*ν* = Δ*E*/*N* when the scattering length is changed by the small amount Δ*a*. Replacing ∂*E*/∂*a* by Δ*E*/Δ*a* in the definition (1), we obtain

4 $$\frac{C}{{C}_{0}}\approx \sqrt{2\pi }\ \frac{m{a}_{z}}{\hslash \bar{n}}\ \frac{{{\Delta }}\nu }{{{\Delta }}a}.$$

To measure the energy change *h*Δ*ν* resulting from a small modification of the scattering length, we take advantage of a particular feature of the ^87^Rb atom: All scattering lengths *a*_*ij*_, with (*i*, *j*) any pair of states belonging to the ground-level manifold, take very similar values^39^. For example, ref. ^40^ predicts *a*_11_ = 100.9 *a*_0_, *a*_22_ = 94.9 *a*_0_ and *a*_12_ = 98.9 *a*_0_, where the indices 1 and 2 refer to the two states $\left|1\right\rangle \equiv \left|F=1,{m}_{z}=0\right\rangle$ and $\left|2\right\rangle \equiv \left|F=2,{m}_{z}=0\right\rangle$ used in this work and *a*_0_ is the Bohr radius. For an isolated atom, this pair of states forms the so-called clock transition at frequency *ν*_0_ ≃ 6.8 GHz, which is insensitive (at first order) to the ambiant magnetic field. Starting from a gas at equilibrium in $\left|1\right\rangle$, we use a Ramsey interferometric scheme to measure the microwave frequency required to transfer all atoms to the state $\left|2\right\rangle$. The displacement of this frequency with respect to *ν*_0_ provides the shift Δ*ν* due to the small modification of scattering length Δ*a* = *a*_22_ − *a*_11_.

The Ramsey scheme consists of two identical microwave pulses, separated by a duration *τ*_1_ = 10 ms. Their duration *τ*_2_ ~ 100 μs is adjusted to have *π*/2 pulses, i.e., each pulse brings an atom initially in $\left|1\right\rangle$ or $\left|2\right\rangle$ into a coherent superposition of these two states with equal weights. Just after the second Ramsey pulse, we measure the 2D spatial density $\bar{n}$ in state $\left|2\right\rangle$ in a disk-shaped region of radius 9 μm, using the absorption of a probe beam nearly resonant with the optical transition connecting $\left|2\right\rangle$ to the excited state $5{P}_{3/2},\ F^{\prime} =3$. We infer from this measurement the fraction of atoms transferred into $\left|2\right\rangle$ by the Ramsey sequence, and we look for the microwave frequency *ν*_*m*_ that maximizes this fraction.

**Fig. 1:**
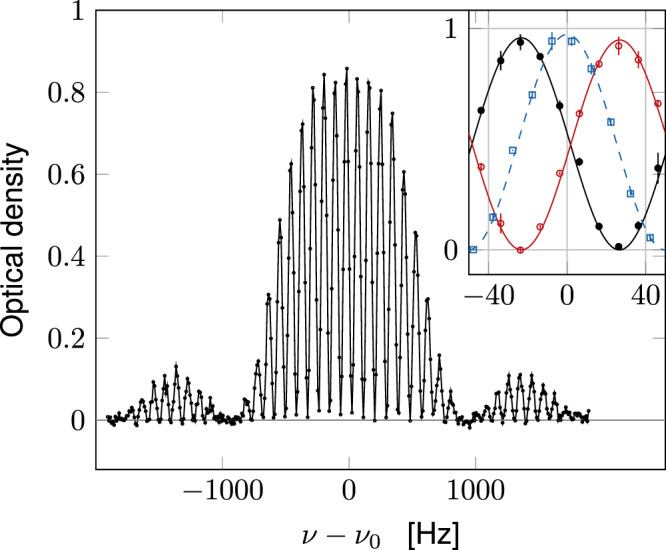
Ramsey signal. Example of an interferometric Ramsey signal showing the optical density of the fraction of the gas in state $\left|2\right\rangle$ after the second Ramsey pulse, as a function of the microwave frequency *ν*. These data were recorded for $\bar{n}\approx 40$ atoms/μm^2^ and *T* ~ 22 nK, *τ*_1_ = 10 ms. Here, *τ*_2_ has been increased to 1 ms to limit the number of fringes for better visibility. Inset. Filled black disks (resp. open red circles): central fringe for atoms in $\left|2\right\rangle$ (resp. $\left|1\right\rangle$) in our standard configuration *τ*_2_ = 0.1 ms. The density in $\left|1\right\rangle$ is obtained by applying a microwave *π*-pulse just before the absorption imaging phase. When atoms are maximally transferred in state $\left|2\right\rangle$, we observe no significant population in state $\left|1\right\rangle$, compatible with a full transfer induced by the Ramsey pulses. Blue squares: single-atom response measured during the ballistic expansion of the cloud by imaging atoms in $\left|2\right\rangle$. The lines in the inset are sinusoidal fits to the data. The vertical error bars of the inset correspond to the standard deviation of the three repetitions made for this measurement.

An example of a spectroscopic signal is shown in Fig. 1. In order to determine the bare transition frequency *ν*_0_, we also perform a similar measurement on a cloud in ballistic expansion, for which the 3D spatial density has been divided by more than 100 and interactions play a negligible role. The uncertainty on the measured interaction-induced shift Δ*ν* = *ν*_*m*_ − *ν*_0_ is on the order of 1 Hz. In principle, the precision of our measurements could be increased further by using a larger *τ*_1_. In practice, however, we have to restrict *τ*_1_ to a value such that the spatial dynamics of the cloud, originating from the non-miscibility of the 1 − 2 mixture (${a}_{12}^{2}\,> \,{a}_{11}{a}_{22}$), plays a negligible role (Supplementary Note 2). We also checked that no detectable spin-changing collisions appear on this time scale: more than 99 % of the atoms stay in the clock state basis. Another limitation to *τ*_1_ comes from atom losses, mostly due to 2-body inelastic processes involving atoms in $\left|2\right\rangle$. For *τ*_1_ = 10 ms, these losses affect <5% of the total population and can be safely neglected.

We see in the inset of Fig. 1 that there indeed exists a frequency *ν*_*m*_ for which nearly all atoms are transferred from $\left|1\right\rangle$ to $\left|2\right\rangle$, so that *E*(*N*, *a*_22_) − *E*(*N*, *a*_11_) = *N* *h*(*ν*_*m*_ − *ν*_0_) (see the Supplementary Note 1 for details). We note that for an interacting system, the existence of such a frequency is by no means to be taken for granted. Here, it is made possible by the fact that the inter-species scattering length *a*_12_ is close to *a*_11_ and *a*_22_. We are thus close to the SU(2) symmetry point where all three scattering lengths coincide. The modeling of the Ramsey process detailed in Supplementary Note 1 shows that this quasi-coincidence allows one to perform a Taylor expansion of the energy *E*(*N*_1_, *N*_2_) (with *N*_1_ + *N*_2_ = *N*) of the mixed system between the two Ramsey pulses, and to expect a quasi-complete rephasing of the contributions of all possible couples (*N*_1_, *N*_2_) for the second Ramsey pulse. The present situation is thus quite different from the one exploited in ref. ^31^, for example, where *a*_11_ and *a*_12_ were vanishingly small. It also differs from the generic situation prevailing in the spectroscopic measurements of Tan’s contact in two-component Fermi gases, where a microwave pulse transfers the atoms to a third, non-interacting^16^ or weakly-interacting state^19^.

**Fig. 2:**
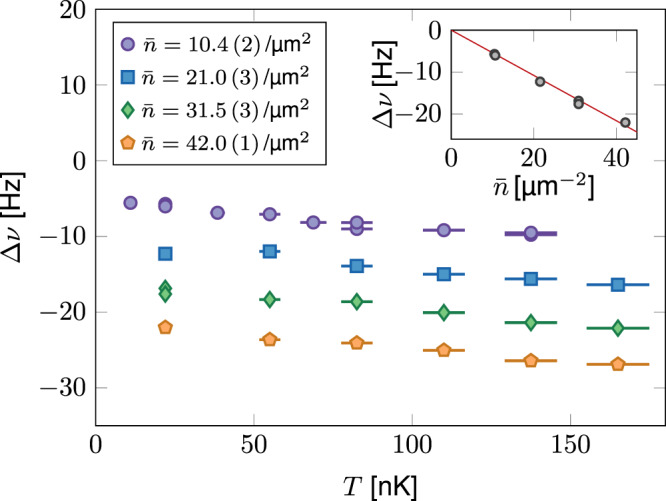
Frequency shift of the resonance. Variations of the shift Δ*ν* with temperature for various 2D spatial densities. The horizontal error bars represent the statistical uncertainty on the temperature calibration, except for the points at very low temperature (10–22 nK). These ultra-cold points are deeply in the Thomas–Fermi regime, where thermometry based on the known equation of state of the gas is not sensitive enough. The temperature is thus inferred from an extrapolation with an evaporation barrier height of the higher temperature points. The error on the frequency measurement is below 1 Hz and is not shown in this graph. Inset: Variations of the shift Δ*ν* with density at low temperature *T* ~ 22 nK, i.e., a strongly degenerate gas. The straight line is the mean-field prediction corresponding to Δ*a* = −5.7 *a*_0_.

We show in Fig. 2 our measurements of the shift Δ*ν* for densities ranging from 10 to 40 atoms/μm^2^, and temperatures from 10 to 170 nK. Since ℏ*ω*_*z*_/*k*_B_ = 210 nK, all data shown here are in the thermodynamic 2D regime *k*_B_*T* < ℏ*ω*_*z*_. More precisely, the population of the ground state of the motion along *z*, estimated from the ideal Bose gas model^41^, is always ≳90 %. All shifts are negative as a consequence of *a*_22_ < *a*_11_: the interaction energy of the gas in state $\left|2\right\rangle$ is slightly lower than in state $\left|1\right\rangle$. For a given density, the measured shift increases in absolute value with temperature. This is in line with the naive prediction of $C\propto g_2(0)$ since density fluctuations are expected to be an increasing function of *T*. Conversely for a given temperature, the shift is (in absolute value) an increasing function of density.

For the lowest temperatures investigated here, we reach the fully condensed regime in spite of the 2D character of the sample, as a result of finite size effects. In this case, the mean-field prediction for the shift reads ${{\Delta }}\nu =\bar{n}\ \hslash \ {{\Delta }}a/(\sqrt{2\pi }\ m{a}_{z})$ [i.e., *C* = *C*_0_ in Eq. (4)]. Our measurements confirm the linear variation of Δ*ν* with $\bar{n}$, as shown in the inset of Fig. 2 summarizing the data for *T* = 22 nK. A linear fit to these data gives Δ*a*/*a*_0_ = −5.7 (1.0) where the error mostly originates from the uncertainty on the density calibration. In the following, we use this value of Δ*a* for inferring the value of *C*/*C*_0_ from the measured shift at any temperature, using Eq. (4). We note that this estimate for Δ*a* is in good agreement with the prediction Δ*a*/*a*_0_ = −6 quoted in ref. ^40^. The first corrections to the linear mean-field prediction were derived (in the 3D case) by Lee, Huang, and Yang in ref. ^42^. For our densities, they have a relative contribution on the order of 5 % of the main signal (Δ*ν* ≲ 1 Hz) (Supplementary Note 3), and their detection is borderline for our current precision.

**Fig. 3:**
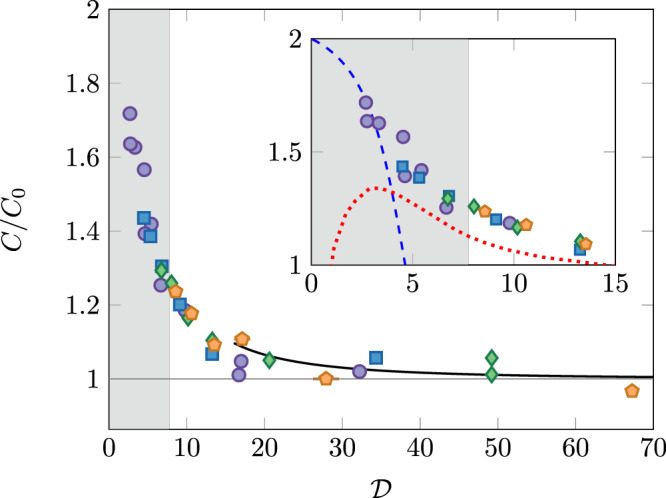
Contact measurement. Variations of the normalized Tan ’s contact *C*/*C*_0_ with the phase-space density ${\mathcal{D}}$. The encoding of the experimental points is the same as in Fig. 2. The colored zone indicates the non-superfluid region, corresponding to ${\mathcal{D}}\,<\,{{\mathcal{D}}}_{{\rm{c}}}\approx 7.7$. The continuous black line shows the prediction derived within the Bogoliubov approximation. Inset: Zoom on the critical region. The dashed blue line is the prediction from ref. ^46^, resulting from a virial expansion for the 2D Bose gas. The dotted red line shows the results of the classical field simulation of ref. ^47^.

We summarize all our data in Fig. 3, where we show the normalized contact *C*/*C*_0_ defined in Eq. (4) as a function of the phase-space density ${\mathcal{D}}$. All data points collapse on a single curve within the experimental error, which is a manifestation of the approximate scale invariance of the Bose gas, valid for a relatively weak interaction strength $\tilde{g}\,\lesssim\,1$^43,44^.

## Discussion

We now compare our results in Fig. 3 to three theoretical predictions. The first one is derived from the Bogoliubov approximation applied to a 2D quasi-condensate^45^. This prediction is expected to be valid only for ${\mathcal{D}}$ notably larger than the phase-space density at the critical point ${{\mathcal{D}}}_{c}$ (see “Methods” section) and it accounts well for our data in the superfluid region. Within this approximation, one can also calculate the two-body correlation function and write it as ${g}_{2}(r)={g}_{2}^{T = 0}(r)+{g}_{2}^{{\rm{thermal}}}(r)$. One can then show the result (Supplementary Note 3)

5 $$\frac{C}{{C}_{0}}=1+{g}_{2}^{{\rm{thermal}}}(0),$$

which provides a quantitative relation between the contact and the pair correlation function, in spite of the already mentioned singularity of ${g}_{2}^{T = 0}(r)$ in *r* = 0.

For low phase-space densities, one can perform a systematic expansion of various thermodynamic functions in powers of the (properly renormalized) interaction strength^46^, and obtain a prediction for *C* (dashed blue line in the inset of Fig. 3). By comparing the 0th, 1st, and 2nd orders of this virial-type expansion, one can estimate that it is valid for ${\mathcal{D}}\,\lesssim\,3$ for our parameters. When ${\mathcal{D}}\to 0$, the result of ref. ^46^ gives *C*/*C*_0_ → 2, which is the expected result for an ideal, non-degenerate Bose gas. The prediction of ref. ^46^ for ${\mathcal{D}} \sim 3$ compares favorably with our results in the weakly degenerate case.

Finally, we also show in Fig. 3 the results of the classical field simulation of ref. ^47^ (red dotted line), which are in principle valid both below and above the critical point. Contrary to the quantum case, this classical analysis does not lead to any singularity for 〈*n*^2^(0)〉, so that we can directly plot this quantity as it is provided in ref. ^47^ in terms of the quasi-condensate density. For our interaction strength, we obtain a non-monotonic variation of *C*. This unexpected behavior, which does not match the experimental observations, probably signals that the present interaction strength $\tilde{g}=0.16$ (see “Methods” section and the Supplementary Note 5) is too large for using these classical field predictions, as already suggested in ref. ^47^.

Using the Ramsey interferometric scheme on a many-body system, we have measured the two-body contact of a 2D Bose gas over a wide range of phase-space densities. We could implement this scheme on our fluid thanks to the similarities of the three scattering lengths in play, *a*_11_, *a*_22_, *a*_12_, corresponding to an approximate SU(2) symmetry for interactions. Our method can be generalized to the strongly interacting case *a*_*ij*_ ≳ *a*_*z*_, as long as a Fano-Feshbach resonance allows one to stay close to the SU(2) point. One could then address the LHY-type corrections at zero temperature^48,49^, the contributions of the weakly-bound dimer state and of three-body contact^13,14^, or the breaking of scale invariance expected at non-zero temperature.

Finally, we note that even for our moderate interaction strength, classical field simulations seem to fail to reproduce our results, although they could properly account for the measurement of the equation of state itself^43,44^. The semi-classical treatment of ref. ^50^ and the quantum Monte Carlo approach of ref. ^51^ (see also ref. ^52^) should provide a reliable path to the modeling of this system. This would be particularly interesting in the vicinity of the BKT transition point where the usual approach based on the *XY* model^53^, which neglects any density fluctuation, does not provide relevant information on Tan’s contact. It would allow one to address the fundamental question raised for example in ref. ^26^, regarding the behavior of the contact $C({\mathcal{D}})$ or its derivatives in the vicinity of the phase transition, and the possibility to signal the position of the critical point either by a singularity or at least a fast variation of Tan’s contact around this point.

## Methods

The preparation and the characterization of our sample have been detailed in^54,55^ and we briefly outline the main properties of the clouds explored in this work. In the *xy* plane, the atoms are confined in a disk of radius 12 μm by a box-like potential, created by a laser beam properly shaped with a digital micromirror device. We use the intensity of this beam, which determines the height of the potential barrier around the disk, as a control parameter for the temperature. The confinement along the *z* direction is provided by a large-period optical lattice, with a single node occupied and *ω*_*z*_/(2*π*) = 4.41 (1) kHz. We set a magnetic field *B* = 0.701 (1) G along the vertical direction *z*, which defines the quantization axis. We use the expression ${{\mathcal{D}}}_{{\rm{c}}}={\mathrm{ln}}\,(380/{\tilde{g}})$ for the phase-space density at the critical point of the superfluid transition^56^. Here, $\tilde{g}=\sqrt{8\pi }\ {a}_{11}/{a}_{z}=0.16$ is the dimensionless interaction strength in 2D, leading to ${{\mathcal{D}}}_{{\rm{c}}}=7.7$. We study Bose gases from the normal regime (${\mathcal{D}}=0.3{{\mathcal{D}}}_{{\rm{c}}}$) to the strongly degenerate, superfluid regime (${\mathcal{D}}\,> \,3{{\mathcal{D}}}_{{\rm{c}}}$).

## Supplementary information

Supplementary Information

## Data Availability

The data sets generated and analyzed during the current study are available from the corresponding author on request.
