# Supplementary material for: Tan’s two-body contact across the superfluid transition of a planar Bose gas
Source: Nat Commun. 2021 Feb 3;12:760. doi: 10.1038/s41467-020-20647-6 (PMC7858573; doi:10.1038/s41467-020-20647-6)
Supplement: Supplementary file 1 — Supplementary Information [file 41467_2020_20647_MOESM1_ESM.pdf]

Supplementary Information for
Tan's two-body contact across the superfluid transition of a planar Bose gas

Y.-Q. Zou, B. Bakkali-Hassani, C. Maury, É. Le Cerf, S. Nascimbene, J. Dalibard, and J. Beugnon
Laboratoire Kastler Brossel, Collège de France, CNRS,
ENS-PSL Research University, Sorbonne Université,
11 Place Marcelin Berthelot, 75005 Paris, France
(Dated: November 19, 2020)

SUPPLEMENTARY NOTE 1: RAMSEY INTERFEROMETRY IN A MANY-BODY SYSTEM CLOSE THE SU(2) SYMMETRY POINT

In this section, we explain why the vicinity of the SU(2) symmetry point where all three scattering lengths are equal ($a_{11} = a_{12} = a_{22}$) allows one to reach a full transfer from $|1\rangle$ to $|2\rangle$ in the Ramsey sequence, in spite of the interactions between the particles. We first explore a two-particle model before turning to the general N-atom case.

The two-particle toy model

The analysis of a system with two particles only, which was pioneered by [1], is often used to gain insight in the N-body case, see e.g. [2, 3] in the context of microwave spectroscopy. Here we consider a pair of atoms each with two internal states $|1\rangle$ and $|2\rangle$ (Fig.1). The initial state of the two-particle system is

$$|11\rangle \otimes |\psi_0\rangle, \tag{1}$$

where $|\psi_0\rangle$ describes the external state of the pair and is symmetric by exchange of the two (bosonic) particles.

The two-body state just after the first $\pi/2$ pulse of the Ramsey sequence is

$$\left[\frac{1}{2}|A\rangle + \frac{1}{\sqrt{2}}|B\rangle + \frac{1}{2}|C\rangle\right] \otimes |\psi_0\rangle. \tag{2}$$

Here we have introduced the three states

$$|A\rangle = |11\rangle \qquad |B\rangle = \frac{1}{\sqrt{2}}\left(|12\rangle + |21\rangle\right) \qquad |C\rangle = |22\rangle \tag{3}$$

which correspond to the triplet states, resulting from the coupling of the two internal states viewed as pseudo-spins 1/2.

The time evolution is described by three operators $\hat{U}_{ij}(t)$ and the state of the system reads at time t:

$$\frac{1}{2}|A\rangle \otimes \left(\hat{U}_{11}(t)|\psi_0\rangle\right) + \frac{1}{\sqrt{2}}|B\rangle \otimes \left(\hat{U}_{12}(t)|\psi_0\rangle\right) + \frac{1}{2}|C\rangle \otimes \left(\hat{U}_{22}(t)|\psi_0\rangle\right). \tag{4}$$

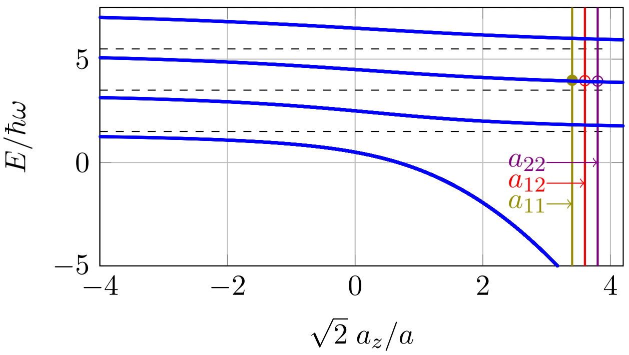

Supplementary Fig. 1. Energy levels of the relative motion of zero angular momentum for a two-particle system in a 3D harmonic trap of frequency ω, as function of the scattering length. To model properly the experimental situation, the characteristic length $a_z = \sqrt{\hbar/m\omega}$ is chosen equal to the interparticle distance $d = \bar{n}^{-1/2}$ ($d \sim a_z \sim 160\,\mathrm{nm}$ for $\bar{n} = 40\,\mu\mathrm{m}^{-2}$, i.e. $\hbar\omega = \hbar^2\bar{n}/m$. Therefore the spacing $\sim 2\hbar\omega$ between adjacent levels is large compared to the interaction energy per particle, $\sim \hbar^2\bar{n}\tilde{g}/m$, since $\tilde{g} \ll 1$. The initial state $|\psi_0\rangle$ considered in the text is marked as $\bullet$ and the two other relevant states $|\phi_0\rangle$ and $|\chi_0\rangle$ are marked as $\circ$. All three scattering lengths a_{11}, a_{12}, a_{22} are close to each other (figure not to scale for actual Rb values).

The action of the second $\pi/2$ pulse at time t reads:

$$|1\rangle \to \frac{1}{\sqrt{2}}\left(|1\rangle + \mathrm{e}^{\mathrm{i}\alpha}|2\rangle\right) \qquad |2\rangle \to \frac{1}{\sqrt{2}}\left(|2\rangle - \mathrm{e}^{-\mathrm{i}\alpha}|1\rangle\right), \tag{5}$$

where $\alpha = 2\pi\nu t$ is the phase of the microwave at this time. After the second pulse, we find the fraction $f_2(t)$ transferred to internal state $|2\rangle$:

$$f_2(t,\alpha) = \frac{1}{2} + \frac{1}{4}\Re\left[\mathrm{e}^{\mathrm{i}\alpha}\left(\langle\hat{U}_{12}^\dagger\hat{U}_{11}\rangle + \langle\hat{U}_{22}^\dagger\hat{U}_{12}\rangle\right)\right], \tag{6}$$

where the averages are taken in state $|\psi_0\rangle$.

The contact is calculated as the derivative with respect to the scattering length of the energy of the system (here the pair of atoms) at constant entropy and in thermal equilibrium. Therefore we can suppose that $|\psi_0\rangle$ is an eigenstate of the two-particle system for the scattering length a_{11} and eventually perform a statistical average over $|\psi_0\rangle$ at the end of the analysis.

To calculate the various matrix elements $\langle \hat{U}_{ij}^\dagger \hat{U}_{kl}\rangle$ entering in the expression (6) of the Ramsey signal, we introduce the eigenbases of the two-particle system for the scattering lengths a_{12} and a_{22}, denoted respectively $\{|\phi_n\rangle\}$ and $\{|\chi_n\rangle\}$. For ^{87}Rb, the three scattering lengths a_{11}, a_{12}, a_{22} are close to each other (5% difference at most). This means that essentially one state contributes to the expansion of $|\psi_0\rangle$ on the basis $\{|\phi_n\rangle\}$ or $\{|\chi_n\rangle\}$:

$$|\psi_0\rangle \approx |\phi_0\rangle \approx |\chi_0\rangle. \tag{7}$$

This validates the assumption of constant entropy needed for the calculation of the contact: the populations of the eigenstates of the external motion of the two-particle system are quasi-unchanged by the Ramsey pulses (Fig.1).

With this assumption, we find

$$\langle \hat{U}_{12}^\dagger \hat{U}_{11}\rangle \approx \mathrm{e}^{\mathrm{i}(E_{12}-E_{11})t/\hbar}, \quad \langle \hat{U}_{22}^\dagger \hat{U}_{12}\rangle \approx \mathrm{e}^{\mathrm{i}(E_{22}-E_{12})t/\hbar} \tag{8}$$

where E_{ij} includes both the single atom energy $\pm h\nu_0/2$ and the interaction energy of the atom pair. The Ramsey signal now reads:

$$\begin{aligned} f_2(t) \approx \frac{1}{2} + \frac{1}{4}\cos\left[\alpha + (E_{12}-E_{11})t/\hbar\right] \\ + \frac{1}{4}\cos\left[\alpha + (E_{22}-E_{12})t/\hbar\right]. \end{aligned} \tag{9}$$

It is maximal for

$$2h\nu = E_{22} - E_{11} \tag{10}$$

as announced in the main text, and it reaches $f_2 = 1$ when a_{12} is equal to the arithmetic mean of a_{11} and a_{22}. These conclusions are unchanged when one subsequently performs a statistical average over $|\psi_0\rangle$.

Achieving a full transfer in the N-body Ramsey sequence

We consider a collection of N two-level atoms with internal states $|1\rangle, |2\rangle$, and we assume that the initial state for the Ramsey sequence corresponds to having all atoms in the internal state $|1\rangle$:

$$|\Psi_0\rangle = \frac{1}{\sqrt{N!}}\left(\hat{a}_1^\dagger\right)^N |0\rangle, \tag{11}$$

with a given external many-body state $|\psi_0\rangle$.

After the first $\pi/2$ pulse, the collective internal state is

$$\begin{aligned} |\Psi_1\rangle &= \frac{1}{\sqrt{2^N\, N!}}\left(\hat{a}_1^\dagger + \hat{a}_2^\dagger\right)^N |0\rangle \\ &= \frac{1}{\sqrt{2^N\, N!}}\sum_{N_1=0}^{N}\binom{N}{N_1}\left(\hat{a}_1^\dagger\right)^{N_1}\left(\hat{a}_2^\dagger\right)^{N_2} |0\rangle. \end{aligned} \tag{12}$$

We denote $E(N_1, N_2)$ the energy of the system with N_1 particles in $|1\rangle$ and $N_2 = N - N_1$ particles in $|2\rangle$. After the evolution for a duration t, the state becomes:

$$\frac{1}{\sqrt{2^N\, N!}}\sum_{N_1=0}^{N}\binom{N}{N_1}\mathrm{e}^{-\mathrm{i}E(N_1,N_2)t/\hbar}\left(\hat{a}_1^\dagger\right)^{N_1}\left(\hat{a}_2^\dagger\right)^{N_2} |0\rangle. \tag{13}$$

The second $\pi/2$ pulse at time t corresponds to

$$\hat{a}_1^\dagger \to \frac{1}{\sqrt{2}}\left(\hat{a}_1^\dagger + \mathrm{e}^{\mathrm{i}\alpha}\hat{a}_2^\dagger\right), \quad \hat{a}_2^\dagger \to \frac{1}{\sqrt{2}}\left(\hat{a}_2^\dagger - \mathrm{e}^{-\mathrm{i}\alpha}\hat{a}_1^\dagger\right), \tag{14}$$

where $\alpha = 2\pi\nu t$ is the phase of the microwave at time t.

In the binomial expansion (12), only the terms (N_1, N_2) that are close to $(N/2, N/2)$ contribute significantly. Therefore we perform a Taylor expansion of the energy of each term at first order in $q = (N_1 - N_2)/2$:

$$E\left(\frac{N}{2}+q, \frac{N}{2}-q\right) \approx E\left(\frac{N}{2}, \frac{N}{2}\right) + (\mu_1 - \mu_2)q \tag{15}$$

where

$$\mu_1 = \left(\frac{\partial E}{\partial N_1}\right)_{N_2}, \quad \mu_2 = \left(\frac{\partial E}{\partial N_2}\right)_{N_1}. \tag{16}$$

With this approximation, each term in the sum (13) has a phase that is proportional to $(N_1 - N_2)t$ and we expect a full transfer to level $|2\rangle$ after the second Ramsey pulse for :

$$h\nu = \mu_1 - \mu_2. \tag{17}$$

Validity of the expansion (15). In order to give a necessary condition on the parameters of the problem for (15) to hold, we consider the $T = 0$ case and use the expression for the mean-field energy:

$$\begin{aligned} E(N_1, N_2) = \frac{1}{2}(N_2 - N_1)h\nu_0 + \\ \frac{\hbar^2}{2mL^2}\left(\tilde{g}_{11}N_1^2 + 2\tilde{g}_{12}N_1N_2 + \tilde{g}_{22}N_2^2\right), \end{aligned} \tag{18}$$

where L^2 is the area of the box confining the gas. One then has the exact result:

$$\begin{aligned} E\left(\frac{N}{2}+q, \frac{N}{2}-q\right) = E\left(\frac{N}{2}, \frac{N}{2}\right) \\ + \left[-h\nu_0 + \frac{\hbar^2}{2m}(\tilde{g}_{11} - \tilde{g}_{22})\bar{n}\right]q \\ + \frac{\hbar^2}{2mL^2}(\tilde{g}_{11} + \tilde{g}_{22} - 2\tilde{g}_{12})q^2. \end{aligned} \tag{19}$$

In practice, we operate the Ramsey scheme in the regime

$$\frac{\hbar t}{2m}|\tilde{g}_{11} - \tilde{g}_{22}|\bar{n} \sim 1 \tag{20}$$

to obtain a good precision on the determination of $\tilde{g}_{11} - \tilde{g}_{22}$. Using the fact that for the binomial distribution, $\langle q^2\rangle = N/4$, we deduce that the contribution of

the last line of (19) [which was omitted in Eq. (15)] can be neglected if:

$$\frac{1}{4}\,|\tilde{g}_{11}+\tilde{g}_{22}-2\tilde{g}_{12}| \lesssim |\tilde{g}_{11}-\tilde{g}_{22}| \tag{21}$$

meaning that the interspecies scattering length a_{12} has to be close to the average of the intraspecies ones, a_{11} and a_{22}. This condition is well fulfilled for ^{87}Rb. Note that the quantity $\tilde{g}_{11}+\tilde{g}_{22}-2\tilde{g}_{12}$ is also known to give the angle of the rotation induced by the one-axis twisting Hamiltonian in binary spinor condensates, which is central to the generation of spin squeezed states [4–7] .

Using the approximate SU(2) symmetry

We have seen above that provided the inequality (21) is satisfied, one can achieve a full transfer from $|1\rangle$ to $|2\rangle$ in the Ramsey sequence operating in the regime (20), provided the microwave frequency is chosen such that

$$h\nu = \left(\frac{\partial E}{\partial N_1}\right)_{N_2} - \left(\frac{\partial E}{\partial N_2}\right)_{N_1}. \tag{22}$$

Here, the energy E is calculated for the parameters $N_1 = N_2 = N/2$ and the 3 scattering lengths a_{11}, a_{12} and a_{22}. Suppose now that all three scattering lengths are close to each other, so that we can expand:

$$\begin{aligned} E(N_1,N_2,a_{11},a_{12},a_{22}) \approx E(N_1,N_2,a,a,a) +\\ (a_{12}-a_{11})\frac{\partial E}{\partial a_{12}} + (a_{22}-a_{11})\frac{\partial E}{\partial a_{22}} \end{aligned} \tag{23}$$

where we have set $a \equiv a_{11}$. The SU(2) symmetry is exact at the point in parameter space where $a_{12} = a_{22} = a$.

We note that:

$$\frac{\partial^2 E}{\partial N_1\,\partial a_{12}}\left(\frac{N}{2},\frac{N}{2},a,a,a\right) = \frac{\partial^2 E}{\partial N_2\,\partial a_{12}}\left(\frac{N}{2},\frac{N}{2},a,a,a\right) \tag{24}$$

so that the term $\propto (a_{12}-a_{11})$ does not contribute to (22). This leads to

$$h\,\Delta\nu = (a_{22}-a_{11})\left[\frac{\partial^2 E}{\partial N_1\,\partial a_{22}} - \frac{\partial^2 E}{\partial N_2\,\partial a_{22}}\right]\left(\frac{N}{2},\frac{N}{2},a,a,a\right) \tag{25}$$

Now, the Hamiltonian of the binary system for a regularized zero-range potential is

$$\hat{H} = \hat{H}_0 + \sum_{i,j} a_{ij}\hat{K}_{ij} \tag{26}$$

where

$$\hat{K}_{ij} = \frac{2\pi\hbar^2}{m}\iint \hat{\psi}_i^\dagger(\boldsymbol{r})\,\hat{\psi}_j^\dagger(\boldsymbol{r}')\,\hat{\delta}(\boldsymbol{r}-\boldsymbol{r}')\,\hat{\psi}_j(\boldsymbol{r}')\,\hat{\psi}_i(\boldsymbol{r})\,\mathrm{d}^3r\,\mathrm{d}^3r'. \tag{27}$$

Hellmann–Feynman theorem thus leads to:

$$h\,\Delta\nu \approx (a_{22}-a_{11})\left[\frac{\partial\langle\hat{K}_{22}\rangle}{\partial N_1} - \frac{\partial\langle\hat{K}_{22}\rangle}{\partial N_2}\right]\left(\frac{N}{2},\frac{N}{2},a,a,a\right) \tag{28}$$

At the SU(2) point, we can connect the two-component system with the single component system with the same scattering length:

$$\langle\hat{K}_{22}\rangle = \frac{N_2^2}{(N_1+N_2)^2}\langle\hat{K}\rangle \tag{29}$$

We then find:

$$N\,h\,\Delta\nu \approx (a_{22}-a_{11})\,\langle\hat{K}\rangle, \tag{30}$$

which also reads, setting $\Delta a = a_{22}-a_{11}$:

$$C = \frac{16\pi^2 m a^2 N}{\hbar}\,\frac{\Delta\nu}{\Delta a}, \tag{31}$$

and which coincides with the expressions (3,5) of the main text.

SUPPLEMENTARY NOTE 2: ROBUSTNESS OF THE RAMSEY INTERFEROMETRY

In this section, we confirm experimentally the robustness of Ramsey interferometry for the determination of the contact. In the previous discussion, we assumed that no spatial dynamics occurs during the Ramsey sequence. In Fig. 2, we confirm experimentally this hypothesis by investigating the variation of the measured frequency shift and contrast of the Ramsey oscillations for :

- Different values of τ_1, the duration of the Ramsey interrogation.
- Different values of τ_2, the duration of the Ramsey $\pi/2$ pulses. The area of the Ramsey pulses is kept constant by adjusting the microwave field power for each value of τ_2.

We observe no significant variation around the values chosen in the main text $\tau_1 = 10\,$ms and $\tau_2 \sim 100\,\mu$s.

SUPPLEMENTARY NOTE 3: CONTACT AND TWO-BODY CORRELATION WITHIN BOGOLIUBOV APPROACH

Bogoliubov operators and contact

We consider a 2D Bose gas confined in a square box $L \times L$ with periodic boundary conditions. We denote $\hat{a}_{\boldsymbol{k}}$ the operator that annihilates a particle with momentum $\hbar\boldsymbol{k}$. We assume that the temperature is low enough

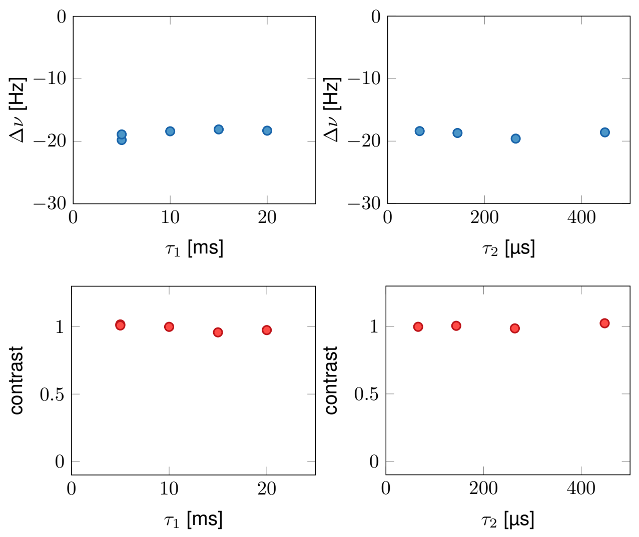

Supplementary Fig. 2. (Top) Frequency shift $\Delta\nu$ as a function of (left) the Ramsey interrogation time τ_1 (for $\tau_2 = 66\,\mu$s) and (right) the Ramsey pulse duration τ_2 (for $\tau_1 = 10\,$ms). (Bottom) Contrast of the Ramsey oscillations as a function of the same parameters. The contrast is defined as $(S_{\max} - S_{\min})/(S_{\max} + S_{\min})$, where S is the fitted sinusoidal variation of the Ramsey oscillations (see the inset of Fig. 1 in the main text).

so that most of the particles accumulate in the ground state of the box $\boldsymbol{k} = 0$. Since the confining box has a finite size, this does not violate Mermin-Wagner theorem, which holds for a gas in the thermodynamic limit. Note that instead of assuming a macroscopic population of $\boldsymbol{k} = 0$, one may also use another version of the Bogoliubov approach in terms of phase and density fluctuations (see e.g. [8]). In that approach, which leads to the same results as the one used here, one assumes that the density fluctuations are small and that the phase fluctuations can be expanded as a Fourier series (no isolated vortex).

The Bogoliubov Hamiltonian is diagonalized by introducing the bosonic operators $\hat{b}_{\boldsymbol{k}} = u_k \hat{a}_{\boldsymbol{k}} - v_k \hat{a}^\dagger_{-\boldsymbol{k}}$ with

$$u_k, v_k = \pm \left[\frac{k^2 + 2\tilde{g}\bar{n}}{2k(k^2 + 4\tilde{g}\bar{n})^{1/2}} \pm \frac{1}{2} \right]^{1/2}, \tag{32}$$

and the energy of the Bogoliubov modes

$$\epsilon_k = \frac{\hbar^2 k}{2m} \left[k^2 + 4\tilde{g}\bar{n} \right]^{1/2}. \tag{33}$$

The Bogoliubov Hamiltonian reads:

$$\hat{H} = E_0 + \sum_{\boldsymbol{k}} \epsilon_k\, \hat{b}^\dagger_{\boldsymbol{k}} \hat{b}_{\boldsymbol{k}}. \tag{34}$$

In the case studied in the paper, where the thickness a_z of the gas is large compared to the scattering length, the ground-state energy E_0 can be estimated by averaging the mean-field 3D result:

$$E_0^{(3\mathrm{D})} = \frac{2\pi\hbar^2 a}{m} n^{(3\mathrm{D})} N \tag{35}$$

over the Gaussian density profile $n^{(3\mathrm{D})}(z) = \bar{n}\, \mathrm{e}^{-z^2/a_z^2} / \sqrt{\pi a_z^2}$ along the z direction:

$$E_0 = \frac{\int E_0^{(3\mathrm{D})}(z)\; n(z)\; \mathrm{d}z}{\int n(z)\; \mathrm{d}z} = \frac{\hbar^2 \tilde{g}}{2m} \bar{n} N \tag{36}$$

The thermal averages are $\langle \hat{b}_{\boldsymbol{k}} \rangle = 0$ and $\langle \hat{b}^\dagger_{\boldsymbol{k}} \hat{b}_{\boldsymbol{k}'} \rangle = \delta_{\boldsymbol{k},\boldsymbol{k}'}\, \mathcal{N}_k$, where $\mathcal{N}_k$ is the Bose–Einstein distribution

$$\mathcal{N}_k = \left[\mathrm{e}^{\epsilon_k / k_\mathrm{B} T} - 1 \right]^{-1}. \tag{37}$$

The internal energy in thermal equilibrium thus reads:

$$E = E_0 + \sum_{\boldsymbol{k}} \epsilon_k \mathcal{N}_k. \tag{38}$$

The contact is by definition proportional to the derivative of this energy with respect to a at constant entropy, i.e. at constant populations $\mathcal{N}_k$ of the modes, which gives:

$$C = C^{T=0} + C^{\mathrm{thermal}} \tag{39}$$

with

$$C^{T=0} = \frac{8\pi m a^2}{\hbar^2} \frac{\partial E_0}{\partial a} = C_0 \tag{40}$$

and

$$\begin{aligned} C^{\mathrm{thermal}} &= \frac{8\pi m a^2}{\hbar^2} \left[\sum_{\boldsymbol{k}} \frac{\partial \epsilon_k}{\partial a} \mathcal{N}_k \right] \\ &= C_0\, \frac{2}{N} \sum_{\boldsymbol{k}} \frac{k}{\sqrt{k^2 + 4\tilde{g}\bar{n}}} \mathcal{N}_k. \end{aligned} \tag{41}$$

Density fluctuations

Average density. The average density of the gas is calculated from $\bar{n} = \langle \hat{n}(\boldsymbol{r}) \rangle$ with $\hat{n}(\boldsymbol{r}) = \hat{\psi}^\dagger(\boldsymbol{r}) \hat{\psi}(\boldsymbol{r})$, and it can be split into a $T = 0$ and a thermal component:

$$\bar{n}^{T=0} = \frac{N_0}{L^2} + \frac{1}{L^2} \sum_{\boldsymbol{k} \neq 0} v_k^2 \tag{42}$$

and

$$\bar{n}^{\mathrm{thermal}} = \frac{1}{L^2} \sum_{\boldsymbol{k} \neq 0} (u_k^2 + v_k^2)\, \mathcal{N}_k. \tag{43}$$

Density correlations. We start from the 4-field correlation function written in normal order $G_2(\boldsymbol{r}) = \langle\hat{\psi}^\dagger(0)\hat{\psi}^\dagger(\boldsymbol{r})\hat{\psi}(\boldsymbol{r})\hat{\psi}(0)\rangle$, which we expand up to first order in $n^{\text{thermal}}/\bar{n}$:

$$G_2(\boldsymbol{r}) = \frac{N_0^2}{L^4} + \frac{N_0}{L^4} \times \sum_{\boldsymbol{k}\neq 0} \mathrm{e}^{\mathrm{i}\boldsymbol{k}\cdot\boldsymbol{r}} \left(\langle\hat{a}_{-\boldsymbol{k}}\hat{a}_{\boldsymbol{k}}\rangle + \langle\hat{a}^\dagger_{-\boldsymbol{k}}\hat{a}^\dagger_{\boldsymbol{k}}\rangle + 2\langle\hat{a}^\dagger_{\boldsymbol{k}}\hat{a}_{\boldsymbol{k}}\rangle\right) + 2\langle\hat{a}^\dagger_{\boldsymbol{k}}\hat{a}_{\boldsymbol{k}}\rangle \tag{44}$$

We can then calculate the g_2 function used in the main text:

$$g_2(\boldsymbol{r}) = \frac{G_2(\boldsymbol{r})}{\bar{n}^2} = g_2^{T=0}(\boldsymbol{r}) + g_2^{\text{thermal}}(\boldsymbol{r}) \tag{45}$$

and we find by at first order in $(\bar{n}-\bar{n}_0)/\bar{n}$ [see e.g. [8]]:

$$g_2^{T=0}(\boldsymbol{r}) = 1 + \frac{2}{N}\sum_{\boldsymbol{k}\neq 0} \mathrm{e}^{\mathrm{i}\boldsymbol{k}\cdot\boldsymbol{r}} v_k(u_k+v_k) \tag{46}$$

and

$$g_2^{\text{thermal}}(\boldsymbol{r}) = \frac{2}{N_0}\sum_{\boldsymbol{k}\neq 0} \mathrm{e}^{\mathrm{i}\boldsymbol{k}\cdot\boldsymbol{r}} \,(u_k+v_k)^2\, \mathcal{N}_k. \tag{47}$$

We notice that

$$(u_k+v_k)^2 = \frac{k}{\sqrt{k^2+4\tilde{g}\bar{n}}} \tag{48}$$

which shows the relation (6) of the main text:

$$\frac{C^{\text{thermal}}}{C_0} = g_2^{\text{thermal}}(0). \tag{49}$$

On the other hand, the integral giving $g_2^{T=0}$ in $\boldsymbol{r}=0$ is UV divergent since $v_k \propto 1/k^2$ and $u_k+v_k \sim 1$ at infinity.

Lee-Huang-Yang (LHY) correction [9]

In 3D and at zero-temperature, the first beyond-mean-field correction to the contact is (see e.g. Eq.(2) in [10])

$$\frac{\delta C}{C} = \frac{5}{2}\times\frac{128}{15\sqrt{\pi}}\sqrt{n^{(3\mathrm{D})}a^3}. \tag{50}$$

In our setup, the average 3D density is $n^{(3\mathrm{D})} = \bar{n}/(a_z\sqrt{2\pi})$. For a 2D density $\bar{n} = 40\,\text{atoms}/\mu\text{m}^2$ and $a_z = 160\,\text{nm}$, this gives $\bar{n}^{(3\mathrm{D})} \approx 1.0\times 10^{14}\,\text{atoms/cm}^3$ and $\delta C/C \approx 4.7\,\%$. For this $\bar{n}$, the mean-field contribution to the contact corresponds to a shift $\Delta\nu = -22\,\text{Hz}$ (Fig. 2 of the main text), and the LHY correction is $\approx 1\,\text{Hz}$, within the uncertainty of our measurements. Note that a more precise theoretical estimate of the LHY correction for our planar geometry should start from the general expression of the ground-state energy of a 2D Bose gas [11–13] and the relation between the 2D scattering length and the 3D one [14].

Estimate for the contribution of the 3-body contact

Using the transition rates derived in [15], Fletcher et al. [3] have shown that the contribution of the 3-body contact to the many-body resonance shift is related to the shift due to the 2-body contact by:

$$\frac{\Delta\nu_3}{\Delta\nu_2} = 5.0\pi^2\, a\,\frac{C_3}{C_2}. \tag{51}$$

Now an estimate of C_3/C_2 for a dilute BEC is provided by [16]:

$$\frac{C_3}{C_2} \sim 0.02\, n^{(3\mathrm{D})}a^2 \tag{52}$$

so that the contribution of the 3-body contact is reduced by a factor $\sim n^{(3\mathrm{D})}a^3 \sim 10^{-5}$ with respect to the contribution of the 2-body contact. Even though the 2D nature of the thermodynamics of our gas may bring some significant corrections to this crude estimate, we can safely assume that effects related to the 3-body contact cannot be detected with our experimental protocol.

SUPPLEMENTARY NOTE 4: VIRIAL EXPANSION AND CONTACT

The 3D case

We consider first a 3D gas with a uniform density $\rho = N/L^3$, where N is the number of particles and L^3 the volume of the gas. The virial expansion consists in expressing the grand potential Ω and the density ρ as series in powers of the fugacity $z = \exp(-\mu/k_\mathrm{B}T)$, where μ is the chemical potential and T the temperature [17]:

$$-\frac{\Omega}{k_\mathrm{B}T} = \frac{L^3}{\lambda^3}\sum_{l=1}^{\infty} b_l(T,a)\,z^l, \qquad \rho\lambda^3 = \sum_{l=1}^{\infty} l\,b_l(T,a)\,z^l \tag{53}$$

with $\lambda = \sqrt{2\pi}\,\hbar/\sqrt{mk_\mathrm{B}T}$. The ideal Boltzmann gas case is obtained by keeping only the first order, which imposes $b_1 = 1$. At order 2, one can eliminate z between these two equations, which gives:

$$\frac{\Omega(L^3,T,\mu,a)}{Nk_\mathrm{B}T} = -1 + b_2(T,a)\,\rho\lambda^3. \tag{54}$$

The free energy $F = \Omega + \mu N$ is at the same order

$$\frac{F(L^3,T,N,a)}{Nk_\mathrm{B}T} = \log(\rho\lambda^3/\mathrm{e}) - b_2(T,a)\,\rho\lambda^3. \tag{55}$$

We consider now the case where the gas occupies an area L^2 in the xy plane and is strongly confined along the z direction, with the density profile $\rho(z) = \bar{n}\exp(-z^2/a_z^2)/(\sqrt{\pi}a_z)$. For our experimental parameters, the 3D scattering length a is much smaller than a_z

so that atomic interactions can still be treated with the 3D formalism. Therefore, we can calculate the contribution of interactions (and Bose statistics) to the free energy $\Delta F = F - F_{\rm Boltzmann}$ by averaging the 3D expression of F over the density profile $\rho(z)$:

$$\frac{\Delta F(L^3,T,N,a)}{N k_{\rm B} T} \approx -b_2(T,a)\lambda^3 \frac{\int \rho^2(z)\ {\rm d}z}{\int \rho(z)\ {\rm d}z} \quad (56)$$

$$= -b_2(T,a)\frac{\bar{n}\lambda^3}{\sqrt{2\pi}a_z}. \quad (57)$$

Then we calculate the contact of the gas using

$$C = \frac{8\pi m a^2}{\hbar^2}\left(\frac{\partial F}{\partial a}\right)_{L^2,T,N}. \quad (58)$$

Using the collisional phase shift $\delta_0(k) \approx -ka$ associated with s-wave scattering, the Beth–Uhlenbeck formula for a Bose gas leads to [17]:

$$b_2(T,a) = \frac{1}{2^{5/2}} - \frac{2a}{\lambda} \quad \Rightarrow \quad \left(\frac{\partial b_2}{\partial a}\right)_T = -\frac{2}{\lambda}, \quad (59)$$

and one finds finally in the weakly degenerate regime

$$C = 2C_0. \quad (60)$$

The 2D case

In [18], H.c Ren gives the result of perturbative thermodynamics applied to a regularized contact potential in 2D. Strictly speaking, this is not a virial expansion, i.e. an expansion in powers of density or fugacity, since the author takes exactly into account all powers of $\bar{n}$ in the ideal gas case.

Starting from the 2D scattering length a_2, Ren introduces the dimensionless coupling

$$\alpha(T) = \frac{1}{\ln\left(\frac{\lambda^2}{2\pi a_2^2}\right) + \gamma} \quad (61)$$

where $\lambda(T)$ is the thermal wavelength and γ the Euler constant, which is related to $\tilde{g}$ by $\tilde{g} \approx 4\pi\alpha$. He then performs a systematic expansion of various thermodynamic functions in powers of α. Note that the T dependence of α explicitly breaks the scale invariance of the problem, as expected after regularization of the contact interaction in 2D. However for our experimental parameters, this T-dependence plays a negligible role.

The value of the free energy F reads at order 2 in α:

$$F(N,L^2,T,a_2) = F_0(N,L^2,T) + \alpha\,\frac{4\pi\hbar^2 N^2}{mL^2} - \alpha^2\,\frac{8\pi L^2\hbar^2}{m\lambda^4}\phi\left[1 - {\rm e}^{-N\lambda^2/L^2}\right], \quad (62)$$

where F_0 is the ideal Bose gas result and where the function $\phi(z)$ is defined by:

$$\phi(z) = B(z) + \frac{1}{2}D(z) \quad (63)$$

with

$$B(z) = \sum_{r,s,t=1}^{\infty} \frac{z^{r+s+t}}{\sqrt{rs(r+t)(s+t)}} \ln\frac{\sqrt{(r+t)(s+t)}+\sqrt{rs}}{\sqrt{(r+t)(s+t)}-\sqrt{rs}} \quad (64)$$

and

$$D(z) = \sum_{r,s=1}^{\infty} \frac{z^{r+s}}{rs}\ln\frac{2rs}{r+s}. \quad (65)$$

Tan's contact

$$C = \frac{8\pi m a^2}{\hbar^2}\left(\frac{\partial F}{\partial a}\right)_{N,L^2,T} \quad (66)$$

can then be calculated using (61) together with the link between the 2D (a_2) and 3D (a) scattering lengths and the size of the ground state along the z direction (a_z) [14, 19, 20]

$$a_2 \approx 1.863\, a_z \exp\left(-\sqrt{\frac{\pi}{2}}\frac{a_z}{a}\right). \quad (67)$$

The result is plotted in Fig. 3 of the article.

SUPPLEMENTARY NOTE 5: CLASSICAL FIELD PREDICTIONS

In the main text we compare our measurements to the results of a classical field simulation [21], which provides the variation of the quasi-condensate density with the phase-space density $\mathcal{D}$. Whereas these simulations lead to reliable results for the equation of state of the 2D Bose gas for our interaction strength $\tilde{g}$ [22], we find an unphysical behaviour when we use them to predict the contact.

First, in the very degenerate regime $\mathcal{D} \gg 1$, they predict $C/C_0 < 1$. This could be possibly corrected by normalizing the classical-field prediction by the Bogoliubov results in this regime, since they should coincide by construction.

More strikingly, the curve does not reach $C/C_0 = 2$ in the non-degenerate regime, and it even displays a non-monotonic variation with $\mathcal{D}$. We show in Fig. 3 that this behaviour is a consequence of the relatively large value of $\tilde{g}$ used here. When lowering $\tilde{g}$, we recover a monotonic variation compatible with the expected non-degenerate limit $C = 2C_0$. This result highlights, as originally stated in Ref. [21], the limited range of applicability of these simulations for determining the quasi-condensate density.

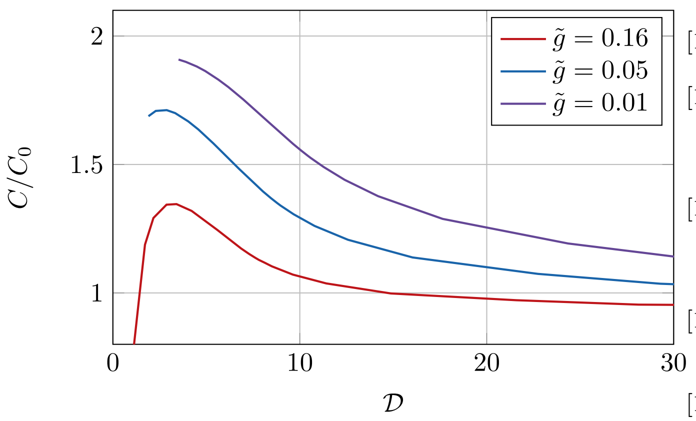

Supplementary Fig. 3. Contact C calculated using the quasi-condensate density given in Ref. [21] (classical field analysis), for different values of the interaction parameter $\tilde{g}$. In the limit $\tilde{g} \to 0$, a monotonic variation of C/C_0 from ~ 2 to ~ 1 is recovered.

SUPPLEMENTARY REFERENCES:

[1] T. Busch, B. G. Englert, K. Rzazewski, and M. Wilkens, "Two cold atoms in a harmonic trap," Foundations of Physics **28**, 549 (1998).

[2] AG Sykes, JP Corson, JP D'Incao, AP Koller, CH Greene, AM Rey, KRA Hazzard, and JL Bohn, "Quenching to unitarity: Quantum dynamics in a three-dimensional bose gas," Phys. Rev. A **89**, 021601 (2014).

[3] R.J. Fletcher, R. Lopes, J. Man, N. Navon, R.P. Smith, M.W. Zwierlein, and Z. Hadzibabic, "Two- and three-body contacts in the unitary Bose gas," Science **355**, 377–380 (2017).

[4] M. Kitagawa and M. Ueda, "Squeezed spin states," Phys. Rev. A **47**, 5138–5143 (1993).

[5] Y. Li, P. Treutlein, J. Reichel, and A. Sinatra, "Spin squeezing in a bimodal condensate: spatial dynamics and particle losses," Eur. Phys. J. B **68**, 365–381 (2009).

[6] C. Gross, T. Zibold, E. Nicklas, J. Esteve, and M.K. Oberthaler, "Nonlinear atom interferometer surpasses classical precision limit," Nature **464**, 1165–1169 (2010).

[7] M.F. Riedel, P. Böhi, Y. Li, T.W. Hänsch, A. Sinatra, and P. Treutlein, "Atom-chip-based generation of entanglement for quantum metrology," Nature **464**, 1170–1173 (2010).

[8] C. Mora and Y. Castin, "Extension of Bogoliubov theory to quasicondensates," Phys. Rev. A **67**, 053615 (2003).

[9] T. D. Lee, K. Huang, and C. N. Yang, "Eigenvalues and eigenfunctions of a Bose system of hard spheres and its low-temperature properties," Phys. Rev. **106**, 1135 (1957).

[10] R.J. Wild, P. Makotyn, J.M. Pino, E.A. Cornell, and D.S. Jin, "Measurements of Tan's contact in an atomic bose-einstein condensate," Phys. Rev. Lett. **108**, 145305 (2012).

[11] M. Schick, "Two-dimensional system of hard-core bosons," Phys. Rev. A **3**, 1067 (1971).

[12] C. Mora and Y. Castin, "Ground state energy of the two-dimensional weakly interacting Bose gas: First correction beyond Bogoliubov theory," Phys. Rev. Lett. **102**, 180404 (2009).

[13] S. Fournais, M. Napiorkowski, R. Reuvers, and J.P. Solovej, "Ground state energy of a dilute two-dimensional Bose gas from the Bogoliubov free energy functional," Journal of Mathematical Physics **60**, 071903 (2019).

[14] D. S. Petrov and G. V. Shlyapnikov, "Interatomic collisions in a tightly confined Bose gas," Phys. Rev. A **64**, 012706 (2001).

[15] E. Braaten, D. Kang, and L. Platter, "Universal relations for identical bosons from three-body physics," Phys. Rev. Lett. **106**, 153005 (2011).

[16] D.H. Smith, E. Braaten, D. Kang, and L. Platter, "Two-body and three-body contacts for identical bosons near unitarity," Phys. Rev. Lett. **112**, 110402 (2014).

[17] K. Huang, *Statistical Mechanics* (Wiley, New York, 1987).

[18] Hai-cang Ren, "The virial expansion of a dilute Bose gas in two dimensions," Journal of statistical physics **114**, 481–501 (2004).

[19] Ludovic Pricoupenko and Maxim Olshanii, "Stability of two-dimensional bose gases in the resonant regime," Journal of Physics B: Atomic, Molecular and Optical Physics **40**, 2065 (2007).

[20] I. Bloch, J. Dalibard, and W. Zwerger, "Many-body physics with ultracold gases," Rev. Mod. Phys. **80**, 885–964 (2008).

[21] N. V. Prokof'ev and B. V. Svistunov, "Two-dimensional weakly interacting Bose gas in the fluctuation region," Phys. Rev. A **66**, 043608 (2002).

[22] T. Yefsah, R. Desbuquois, L. Chomaz, K. J. Günter, and J. Dalibard, "Exploring the thermodynamics of a two-dimensional Bose gas," Phys. Rev. Lett. **107**, 130401 (2011).
